# Difference in outcome event coverage between insurance-based and hospital-based databases: a methodological study of diabetes drug use and cardiovascular events in Japan

**DOI:** 10.3389/fphar.2025.1642522

**Published:** 2025-09-16

**Authors:** Takashi Ando, Tomoaki Hasegawa, Chieko Ishiguro, Jun Komiyama, Toshiki Kuno, Masao Iwagami

**Affiliations:** ^1^ Department of Digital Health, Institute of Medicine, University of Tsukuba, Ibaraki, Japan; ^2^ Laboratory of Clinical Epidemiology, Department of Data Science, Center for Clinical Sciences, Japan Institute for Health Security, Tokyo, Japan; ^3^ Department of Health Services Research, Institute of Medicine, University of Tsukuba, Tsukuba, Ibaraki, Japan; ^4^ Health Services Research and Development Center, University of Tsukuba, Tsukuba, Ibaraki, Japan; ^5^ Cardiology Division, Massachusetts General Hospital, Harvard Medical School, Boston, MA, United States; ^6^ Division of Cardiology, Beth Israel Deaconess Medical Center, Harvard Medical School, Boston, MA, United States

**Keywords:** pharmacoepidemiology, administrative claims database, hospital database, diabetes, dipeptidyl peptidase-4 inhibitors, sodium-glucose cotransporter-2 inhibitors

## Abstract

**Introduction:**

In countries with unrestricted access to healthcare, such as Japan, patients may initiate a drug at a clinic or hospital and then may visit another hospital when outcome events occur. Theoretically, an insurance-based database can capture all outcomes, whereas a hospital-based database can only capture outcomes when patients visit that hospital. We examined the difference in outcome event coverage between insurance-based and hospital-based databases in Japan, and its impact on pharmacoepidemiology studies, using diabetes drug use and cardiovascular events as an example.

**Methods:**

Using the JMDC payer database, we identified new users of sodium-glucose cotransporter-2 (SGLT2) inhibitors or dipeptidyl peptidase-4 (DPP-4) inhibitors as the first choice of treatment for type 2 diabetes. Composite outcome was defined as the first hospitalization with a diagnosis of heart failure, stroke, or myocardial infarction. Among patients who initiated drug use at hospitals, we estimated the proportion of events captured in the same hospital among all events recorded in the insurance data. Subsequently, considering a hypothetical hospital-based database study (in which outcome events could only be captured in the same hospital), we estimated an adjusted hazard ratio (aHR) for SGLT2 *versus* DPP-4 inhibitors.

**Results:**

There were 72,556 and 39,214 new users of DPP-4 and SGLT2 inhibitors, respectively, with no history of cardiovascular events, including 18,325 and 9,478 who initiated treatments at hospitals, respectively. Among the 18,325 patients who initiated DPP-4 inhibitors, 195 events occurred, of which 94 (48%) could be captured in the same hospital. Among the 9,478 patients who initiated SGLT-2 inhibitors, 89 events occurred, of which 40 (45%) could be captured in the same hospital. The aHR (95% confidence interval) was 0.74 (0.49–1.12) in the hypothetical hospital-based database study, whereas it was 0.88 (0.64–1.21) in the insurance-based analysis. A sensitivity analysis restricted to hospitals in the Japanese Diagnosis Procedure Combination (DPC) system showed that the percentage exceeded 50% for both the composite and individual disease events.

**Discussion:**

This Japanese study revealed that nearly half (over half when restricted to DPC hospitals) of cardiovascular events were captured in the same hospital where the diabetes drug was initiated.

## 1 Introduction

Over the past decades, an increasing number of pharmacoepidemiology studies have been conducted worldwide, utilizing databases of routinely collected healthcare data, such as administrative claims data and electronic health records of clinics and/or hospitals. Routinely collected healthcare databases can be classified as (i) integrated healthcare databases (consisting of any available healthcare records, which are linked with personal identifiers), (ii) primary care-based databases (consisting of records from general practitioners or clinics), (iii) hospital-based databases (consisting of records from hospitals), and (iv) administrative claims databases (consisting of claims data of people with relevant insurance) (Carrero et al., 2023). Each country or region may have some of these databases, depending on the underlying healthcare and insurance system. For example, in Japan, there are mainly two types of databases (Kumamaru et al., 2024): hospital-based databases such as the Diagnosis Procedure Combination (DPC) database (Yasunaga, 2024a) and the Medical Information Database NETwork (MID-NET^®^) (Yamaguchi et al., 2019), as well as many Japanese disease registries (Clinical Innovation Network); and administrative claims databases or insurance-based databases, such as the National Database of Health Insurance Claims (Yasunaga, 2024b) and the JMDC payer database (Nagai et al., 2021).

In pharmacoepidemiology studies, specifically cohort studies comparing the use of two or more drugs for the incidence of outcome events associated with drug safety or effectiveness, the traceability of the studied database (i.e., to what extent information can be comprehensively captured for each patient) is important (Carrero et al., 2023). Notably, in hospital-based databases, unless the data is linked to other data sources (such as insurance-based claims data and follow-up surveys by telephone call), the data are recorded only when a patient visits the same hospital. Such data fragmentation may cause misclassification of outcome status and (informative) loss-to-follow-up, potentially leading to biased study results (Carrero et al., 2023). Despite these potential concerns, Japanese hospital-based databases have been actively used for international collaborative research, together with other types of databases in other countries (Kohsaka et al., 2020; Khunti et al., 2021; Kosiborod et al., 2018; Heerspink et al., 2020; Kohsaka et al., 2021; Lam et al., 2021; Goh et al., 2023; Vistisen et al., 2023; Karasik et al., 2023; Sheu et al., 2022; Seino et al., 2021).

In Japan, unrestricted access to healthcare is allowed under the universal healthcare system (Ikegami et al., 2011), and patients can visit any medical institution, either a clinic or hospital. To encourage patients with mild chronic diseases (e.g., diabetes) to visit clinics, some Japanese hospitals have introduced a system of additional fees for patients who directly visit large hospitals without referral letters. However, some patients prefer to visit large hospitals directly for specialist consultations. This means that pharmacoepidemiology studies on chronic diseases (e.g., diabetes) can be performed using Japanese hospital-based databases (Kohsaka et al., 2020; Khunti et al., 2021; Kosiborod et al., 2018; Heerspink et al., 2020; Kohsaka et al., 2021; Lam et al., 2021; Goh et al., 2023; Vistisen et al., 2023; Karasik et al., 2023; Sheu et al., 2022; Seino et al., 2021). However, patients who initiate a drug in one hospital may visit other hospitals when outcome events occur. To the best of our knowledge, no Japanese study has been conducted to assess the extent to which the outcome events of patients who initiate a drug in a hospital can be captured in the same hospital, and its impact on pharmacoepidemiology studies.

In the present study, assuming a pharmacoepidemiology research comparing new users of dipeptidyl peptidase-4 (DPP-4) inhibitors and sodium-glucose cotransporter-2 (SGLT2) inhibitors for cardiovascular events, we aimed to assess the concordance or discordance of hospitals where drug use was initiated and where outcome events were captured using an insurance-based database (in which prescriptions and outcomes are recorded, with medical institution IDs of each visit). We also assessed how this impacts a hypothetical hospital-based database study (in which prescriptions and outcomes are captured only in the same hospital where drug treatment was initiated). Both DPP-4 and SGLT2 inhibitors have been selected as the first choice for type 2 diabetes in Japanese clinical practice, and they are appropriate active comparators in the Japanese context.

## 2 Materials and methods

### 2.1 Data source

The JMDC payer database has been detailed previously (Nagai et al., 2021). JMDC Inc. (formerly Japan Medical Data Center Co. until 2018) has obtained individual medical claims and annual health checkup data from participating associations within the Japanese Health Insurance Societies for employee insurance, which cover companies with ≥700 regular employees or groups of companies with a total of ≥3,000 regular employees, as well as their dependents aged <75 years. Since 2005, the number of individuals included in the JMDC payer database has consistently increased, reaching a cumulative total of over 20 million by the end of 2024. The JMDC payer database includes all monthly claims for outpatient and inpatient diagnoses recorded using the original Japanese diagnosis codes, corresponding to the International Classification of Diseases and Related Health Problems, 10th Revision (ICD-10) codes. The database also includes data on medical procedures and drug prescription and dispensation recorded using the original Japanese drug codes and product names, as well as the World Health Organization Anatomical Therapeutic Chemical (WHO-ATC) classification. In addition, the database includes anonymized IDs of medical institutions, with which we could discern which drug was prescribed by which medical institution, as well as the type of medical institution (clinic or hospital). Moreover, the JMDC payer database includes the results of annual health checks provided by health insurance associations, such as hemoglobin A1c (HbA1c), body mass index (BMI), and smoking status.

In this study, we used the most recent dataset, extracted in December 2024, which includes data from January 2005 to November 2024. The data used in this study were anonymized and processed anonymously by JMDC Inc.

### 2.2 Ethics statement

The study was conducted in accordance with the principles of the Declaration of Helsinki. The study was approved by the Ethics Committee of the Institute of Medicine, University of Tsukuba (approval number: 2,127). The need for informed consent was waived because of the anonymous nature of the data.

### 2.3 Study population and exposure

We identified new users of any diabetes drugs (WHO-ATC code A10), defined as those who did not receive prescription or dispensation for any of these drugs for 6 months since registration to the JMDC payer database and then initiating one of these drugs. Among these, we identified those initiating DPP-4 or SGLT2 inhibitors (WHO-ATC codes A10BH or A10BK, respectively). The first “dispensation date” was determined as the day the patient initiated the studied drug (“day 0”).

We then excluded (i) patients who did not receive diabetes diagnoses (ICD-10 codes E11–E14) on day 0 or before, (ii) patients with type 1 diabetes (ICD-10 codes E10) on day 0 or before, (iii) patients who initiated the studied drug at inpatient setting, (iv) patients who started another class of diabetes drugs other than the studied drug (meaning that only new users of DPP-4 inhibitors or SGLT2 inhibitors as the first choice of treatments for type 2 diabetes would be included in the present study), and (v) patients with no follow-up because they initiated the studied drug on their last day according to the JMDC payer database. In addition, for composite and individual outcome events (as shown below), each analysis excluded patients with a history of that outcome, recorded as either an inpatient or outpatient diagnosis (which could suggest a history even before the patient was registered to the JMDC payer database), if its start date of consultation (“shinryo-kaishi-nengappi” in the Japanese claims data) was on day 0 or before.

### 2.4 Outcomes

Considering the number of outcome events (shown later) in the main analysis, composite outcome was defined as the first hospitalization with a diagnosis of (i) heart failure (ICD-10 codes I50, I11.0, I13.0, or I13.2), (ii) stroke (ICD-10 codes I60–I63), or (iii) myocardial infarction (ICD-10 codes I21–I23) regardless of code position. In a Japanese validation study evaluating similar ICD-10 codes in patients with type 2 diabetes among over 200 hospitals, the positive predictive value (PPV) was over 95.7% for heart failure, nearly 88.9% for stroke, and 78.7% for myocardial infarction (Ono et al., 2020). In another validation study evaluating the ICD-10 codes in the DPC database among four hospitals, the sensitivity, specificity, and PPV was 68.8%, 97.5%, and 75.9%, respectively, for congestive heart failure; 50.0%, 98.9%, and 86.4%, respectively, for cerebrovascular disease; and 52.2%, 99.7%, and 92.3%, respectively, for myocardial infarction (Yamana et al., 2017).

We determined whether the medical institution ID recorded for the diagnosis was the same as or different from the medical institution ID recorded for the initiation of the studied drug.

The analysis was repeated for each disease event: heart failure, stroke, and myocardial infarction.

### 2.5 Follow-up

Follow-up started on day 0 and ended at the earliest of the following: incidence of outcome events (i.e., composite event in the main analysis and each disease event in additional analysis); withdrawal from the JMDC payer database (suggesting loss of employee insurance or withdrawal of the health insurance association from contributing to the JMDC payer database); end of November 2024; start or switch to another diabetes drug (because subsequent outcomes may be due to either the initial drug or another drug); or timing of discontinuation of the initiated drug. To define the timing of discontinuation of the initiated drug, we assumed that the initiated drug was continued if the next dispensation was observed within the end of the current dispensation (that is, the calendar date of dispensation plus the number of days dispensed) plus 60 days as the gap period for potential stockpiling. If the next dispensation was not observed during this period, we assumed that the initiated drug was discontinued at the end of the last dispensation plus 60 days.

### 2.6 Covariates

As potential confounding factors, in addition to age, sex, and year of drug initiation, we identified drug prescription and dispensation for hypertension (WHO-ATC codes C02, C03, C07, C08, or C09), dyslipidemia (WHO-ATC codes C10), and hyperuricemia (WHO-ATC codes M04) on day 0 or before. From the annual health check-up data, we identified the most recent HbA1c levels, BMI, and smoking status prior to drug initiation. Some patients had missing values for these health check-up variables, who were excluded from the last model (model 4) adjusting for these variables (as shown below).

### 2.7 Statistical analysis

Baseline patient characteristics were described by initiated drug type (DPP-4 or SGLT2 inhibitors) and medical institution type (clinic or hospital), with their p-values (by t-tests or chi square tests as appropriate) and standardized mean differences.

Focusing on patients who started to use the studied drug at hospitals, we estimated the proportion of events captured in the same hospital among all events recorded in the insurance-based claims data. In addition, to visualize the temporal trend, we plotted the total number of events as well as the number and proportion of events captured in the same hospital, by year of outcome event occurrence.

In the entire JMDC payer database and by type of medical institution (clinic or hospital) where the drug was initiated, we estimated the incidence rates of the outcome events in each group and conducted Cox regression analyses to compare new users of SGLT-2 inhibitors with new users of DPP4 inhibitors (reference group) regarding the incidences of these events. We estimated crude hazard ratios (HRs) and adjusted HRs (aHRs) using four models: model one adjusted for age and sex; model two adjusted for age, sex, year and medication for hypertension, dyslipidemia, and hyperuricemia; model three was based on an inverse probability weighting of propensity score calculated from age, sex, year and medication for hypertension, dyslipidemia, and hyperuricemia to estimate an average treatment effect; and model four adjusted for age, sex, year and medication for hypertension, dyslipidemia, and hyperuricemia, HbA1c level, BMI, and smoking status, as a complete case analysis.

Finally, considering a hypothetical hospital-based database study (in which prescriptions and outcome events could be captured only in the same hospital), we repeated the aforementioned analysis but considered the outcome event only when it was recorded in the same hospital. We used only information on prescriptions and outcome events recorded at the same hospital where the studied drug was initiated. The result of model three was compared to that of insurance-based analysis. Model four was not constructed owing to the limited number of outcome events (as shown later) and because the hypothetical hospital-based database study would not have included annual health checkup data from the community.

As a sensitivity analysis, we focused only on hospitals participating in the Japanese DPC system, established by the Ministry of Health, Labour and Welfare (MHLW) in Japan in 2002. The DPC system is a case-mix patient classification framework linked to a per-diem lump-sum payment system for inpatients (Yasunaga, 2024a). This is because several hospital-based databases in Japan, such as the DPC database and MID-NET^®^, consist of only DPC hospitals.

All analyses were performed using STATA version 17 software (StataCorp, College Station, TX, USA).

## 3 Results

### 3.1 Patient characteristics

**FIGURE 1:**
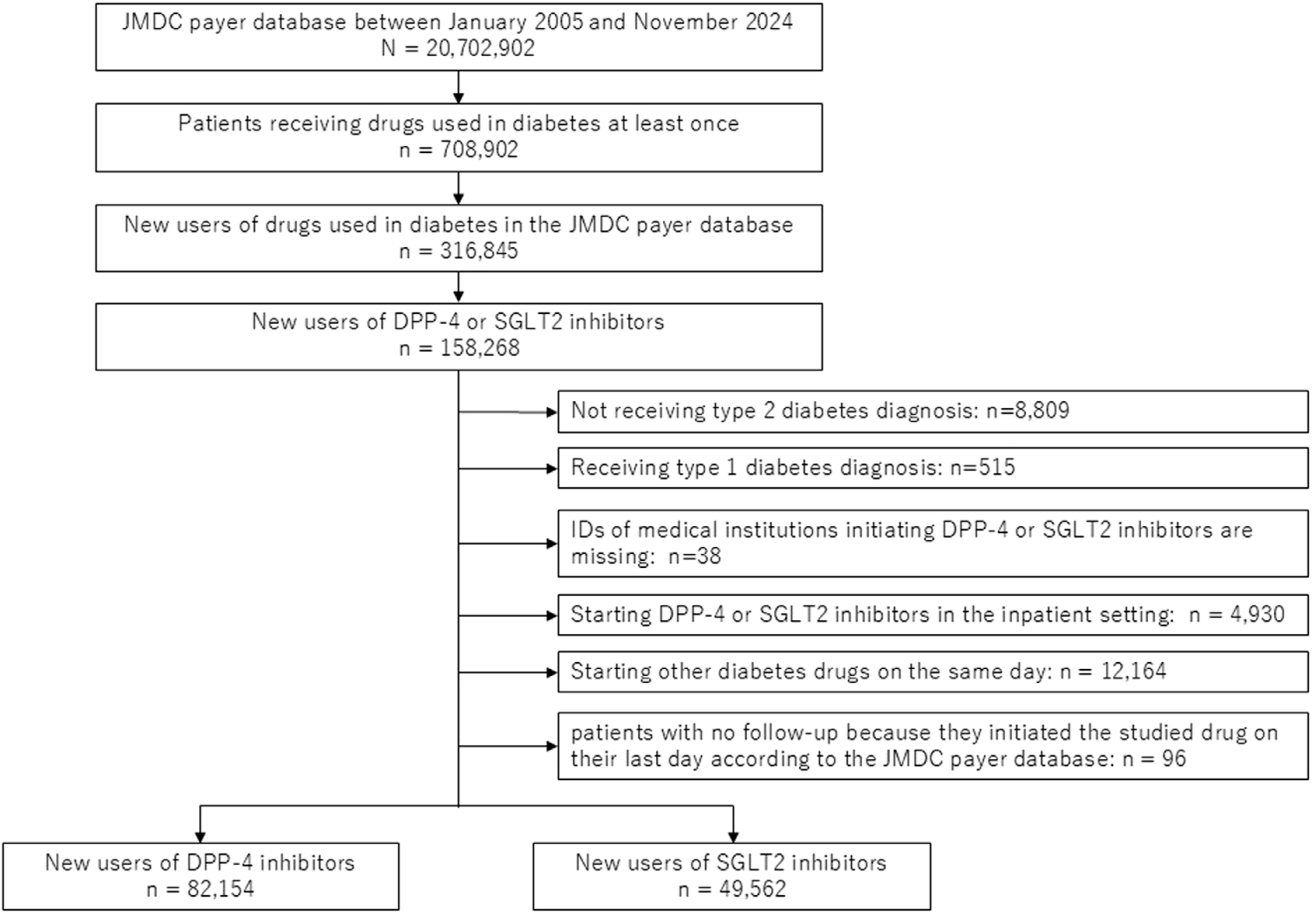
Flow chart of the study. DPP-4, dipeptidyl peptidase-4; SGLT2, sodium-glucose cotransporter-2.

Among over 20 million people in the JMDC payer database, we identified 158,268 new users of DPP-4 or SGLT2 inhibitors (Figure 1). After applying the exclusion criteria, there were 82,154 new users of DPP-4 inhibitors (including 60,028 and 22,126 patients who initiated treatment at clinics and hospitals, respectively) and 49,562 new users of SGLT2 inhibitors (including 35,111 and 14,451 patients who initiated treatment at clinics and hospitals, respectively). Comparing baseline characteristics by drug type, new users of SGLT2 inhibitors were slightly younger; initiated the drug in more recent years; had smaller Hb1c level and higher BMI; were more likely to use drugs for hypertension, dyslipidemia, and hyperuricemia; and were more likely to have a history of heart failure and myocardial infarction (Table 1). Comparing outcomes by medical institution type, patients who initiated treatment at hospitals were more likely to have a history of heart failure, stroke, and myocardial infarction than those who initiated treatment at clinics (Supplementary Table S1).

**TABLE 1:** Baseline characteristics of participants.

Variables	New users of DPP-4 inhibitors N = 82,154	New users of SGLT2 inhibitors N = 49,562	P-value	SMD
Type of medical institutions (%)			<0.001	
Clinics	60,028 (73.1)	35,111 (70.8)		0.050
Hospitals	22,126 (26.9)	14,451 (29.2)		0.050
Age, mean (SD)	54.6 (9.8)	52.5 (10.1)	<0.001	0.211
Sex, n (%)			0.061	
Men	59,076 (71.9)	35,876 (72.4)		0.011
Women	23,078 (28.1)	13,686 (27.6)		0.011
Year, n (%)			<0.001	
2009–2013	5,895 (7.2)	0 (0)		0.393
2014	3,256 (4.0)	210 (0.4)		0.243
2015	3,334 (4.1)	422 (0.9)		0.208
2016	4,602 (5.6)	1,000 (2.0)		0.188
2017	6,423 (7.8)	1,779 (3.6)		0.183
2018	7,919 (9.6)	2,948 (5.9)		0.138
2019	8,909 (10.8)	4,016 (8.1)		0.094
2020	9,275 (11.3)	4,881 (9.8)		0.047
2021	10,373 (12.6)	7,876 (15.9)		0.093
2022	9,018 (11.0)	9,325 (18.8)		0.221
2023	9,303 (11.3)	11,619 (23.4)		0.324
2024	3,847 (4.7)	5,486 (11.1)		0.239
HbA1c, mean (SD)	7.5 (1.5)	6.9 (1.4)	<0.001	0.414
Missing, n (%)	19,934 (24.3)	9,280 (18.7)	<0.001	0.135
Body mass index, mean (SD)	26.9 (4.7)	29.1 (5.3)	<0.001	0.439
Missing, n (%)	15,570 (19.0)	7,463 (15.1)	<0.001	0.104
Smoking history, n (%)			<0.001	
Yes	25,795 (31.4)	14,840 (29.9)		0.032
No	40,412 (49.2)	27,032 (54.5)		0.107
Missing	15,947 (19.4)	7,690 (15.5)		0.103
Prescriptions, n (%)				
Drugs for hypertension	41,395 (50.4)	30,363 (61.3)	<0.001	0.220
Drugs for dyslipidemia	35,965 (43.8)	25,209 (50.9)	<0.001	0.142
Drugs for hyperuricemia	11,950 (14.5)	11,702 (23.6)	<0.001	0.232
Previous diagnosis history, n (%)				
Heart failure	6,868 (8.4)	9,126 (18.4)	<0.001	0.299
Stroke	3,096 (3.8)	1,695 (3.4)	0.001	0.019
Myocardial infarction	841 (1.0)	1,200 (2.4)	<0.001	0.108

SD, standard deviation; SMD, standardized mean difference; DPP-4, dipeptidyl peptidase-4, SGLT2 = sodium-glucose cotransporter-2.

### 3.2 Composite outcome

Regarding composite outcome in the main analysis, after excluding patients with a history of heart failure, stroke, and myocardial infarction, 72,556 and 39,214 new users of DPP-4 and SGLT2 inhibitors, including 18,325 and 9,478 who initiated drug use at hospitals, respectively, were analyzed.

**FIGURE 2:**
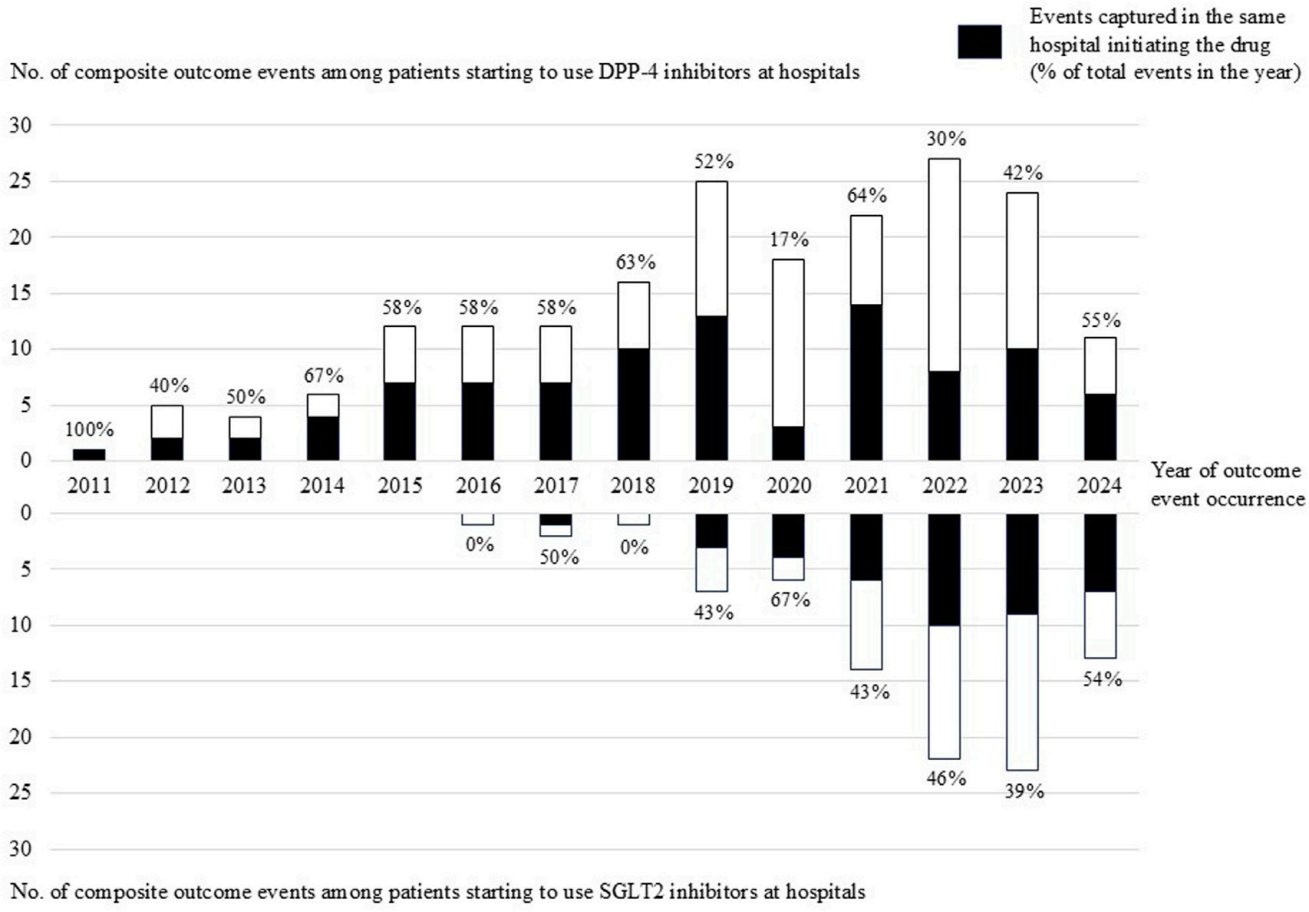
Number of composite outcome events among patients initiating DPP-4 inhibitors or SGLT2 inhibitors at hospitals by year of outcome event occurrence.

Among the 18,325 patients who initiated DPP-4 inhibitors at hospitals, 195 events occurred, of which 94 (48%) were captured in the same hospital (Table 2). Among the 9,478 patients who initiated SGLT-2 inhibitors at hospitals, 89 events occurred, of which 40 (45%) were captured in the same hospital. By year of outcome occurrence, some fluctuations were observed in the proportions of outcome events captured at the same hospital, especially during the COVID-19 pandemic (Figure 2). Table 2 shows the sensitivity analysis restricted to DPC hospitals. Among 11,278 patients who initiated DPP-4 inhibitors at DPC hospitals, 126 events occurred, of which 73 (58%) were captured in the same DPC hospital. Among 6,181 patients who initiated SGLT-2 inhibitors at DPC hospitals, 60 events occurred, of which 32 (53%) were captured in the same DPC hospital.

**TABLE 2:** Number of outcome events in the insurance-based claims data and those captured in the same medical institution.

Drugs	Type of medical institutions starting the drug	No. of new users with no history of that outcome	No. of outcome events in the insurance-based claims data (%)	No. of outcomes events captured in the same medical institution (%)
Composite outcome				
DPP-4 inhibitors	Clinics	54,231	390	n/a
	Hospitals	18,325	195 (100%)	94 (48%)
	DPC hospitals only (sensitivity analysis)	11,278	126 (100%)	73 (58%)
SGLT-2 inhibitors	Clinics	29,736	173	n/a
	Hospitals	9,478	89 (100%)	40 (45%)
	DPC hospitals only (sensitivity analysis)	6,181	60 (100%)	32 (53%)
Heart failure				
DPP-4 inhibitors	Clinics	55,909	220	n/a
	Hospitals	19,377	138 (100%)	68 (49%)
	DPC hospitals only (sensitivity analysis)	11,991	92 (100%)	49 (53%)
SGLT-2 inhibitors	Clinics	30,519	98	n/a
	Hospitals	9,917	59 (100%)	29 (49%)
	DPC hospitals only (sensitivity analysis)	6,509	44 (100%)	25 (57%)
Stroke				
DPP-4 inhibitors	Clinics	58,155	166	n/a
	Hospitals	20,903	78 (100%)	34 (44%)
	DPC hospitals only (sensitivity analysis)	13,196	51 (100%)	30 (59%)
SGLT-2 inhibitors	Clinics	34,116	74	n/a
	Hospitals	13,751	55 (100%)	30 (55%)
	DPC hospitals only (sensitivity analysis)	9,817	41 (100%)	24 (59%)
Myocardial infarction				
DPP-4 inhibitors	Clinics	59,580	104	n/a
	Hospitals	21,733	48 (100%)	18 (38%)
	DPC hospitals only (sensitivity analysis)	13,736	28 (100%)	16 (57%)
SGLT-2 inhibitors	Clinics	34,611	53	n/a
	Hospitals	13,751	37 (100%)	14 (38%)
	DPC hospitals only (sensitivity analysis)	9,762	27 (100%)	14 (52%)

DPC, diagnosis procedure combination; DPP-4, dipeptidyl peptidase-4, SGLT2 = sodium-glucose cotransporter-2.

### 3.3 Each disease outcome

Regarding each disease outcome, among patients who initiated DPP-4 and SGLT2 inhibitors at hospitals with no history of that disease, the outcome event coverages in the same hospital were 49% (68/138) and 49% (29/59) for heart failure, 44% (34/78) and 55% (30/55) for stroke, and 38% (18/48) and 38% (14/37) for myocardial infarction, respectively. However, in the sensitivity analysis restricted to DPC hospitals, all percentages were higher, exceeding 50%: 53% (49/92) and 57% (25/44) for heart failure, 59% (30/51) and 59% (24/41) for stroke, and 57% (16/28) and 52% (14/27) for myocardial infarction, respectively.

### 3.4 The insurance-based analysis

In the JMDC payer database, overall (i.e., combining patients who initiated the studied drugs at clinics and hospitals), the incidence rate (95% confidence interval) of the composite outcome was 6.5 (6.0–7.1) and 5.7 (5.1–6.5) among new users of DPP-4 and SGLT2 inhibitors, respectively. The crude HR (SGLT2 vs DPP-4 inhibitors as a reference group) was 0.88 (0.76–1.02) and the aHR in model 3 (based on an inverse probability weighting of propensity score calculated from age, sex, year, and drugs for hypertension, dyslipidemia, and hyperuricemia) was 0.94 (0.80–1.11) (Table 3). By type of medical institution, the incidence rates were higher among those who initiated the studied drugs at hospitals than among those who initiated them at clinics. Among patients who initiated DPP-4 and SGLT2 inhibitors at hospitals, the incidence rates of the composite outcome (captured in the insurance-based claims data) was 9.6 (8.3–11.0) and 8.2 (6.7–10.1), respectively. The crude and adjusted HRs tended to be slightly lower among those who initiated the studied drugs at hospitals than among those who initiated them at clinics (Table 3). Among new users at hospitals, the crude HR was 0.86 (0.67–1.10) and the aHR in model three was 0.88 (0.64–1.21).

**TABLE 3:** Incidence rates and hazard ratios comparing new users of DPP-4 and SGLT2 inhibitors for the composite outcome.

Type of analysis	DPP-4 inhibitors		SGLT2 inhibitors		Hazard ratio (95% CI) comparing new users of SGLT2 inhibitors and DPP-4 inhibitors (references group)				
	No. of events/ No. of new users with no history of that outcome	Incidence rate (per 1000 PY) (95% CI)	No. of events/ No. of new users with no history of that outcome	Incidence rate (per 1000 PY) (95% CI)	Unadjusted	Model 1*	Model 2**	Model 3***	Model 4****
Overall in the JMDC payer database	585/72,556	6.5 (6.0–7.1)	262/39,214	5.7 (5.1–6.5)	0.88 (0.76–1.02)	1.02 (0.88–1.18)	0.98 (0.84–1.15)	0.94 (0.80–1.11)	1.04 (0.87–1.25)
Subgroup analysis of new users at clinics in the JMDC payer database	390/54,231	5.6 (5.1–6.2)	173/29,736	5.0 (4.3–5.8)	0.89 (0.75–1.07)	1.03 (0.85–1.23)	0.98 (0.81–1.19)	0.96 (0.79–1.18)	1.00 (0.80–1.25)
Subgroup analysis of new users at hospitals in the JMDC payer database	195/18,325	9.6 (8.3–11.0)	89/9,478	8.2 (6.7–10.1)	0.86 (0.67–1.10)	0.97 (0.75–1.26)	0.94 (0.71–1.24)	0.88 (0.64–1.21)	1.11 (0.80–1.54)
Hypothetical hospital-based analysis	86/18,325	4.7 (3.8–5.8)	39/9,478	3.9 (2.8–5.3)	0.84 (0.57–1.22)	0.93 (0.64–1.37)	0.90 (0.60–1.37)	0.74 (0.49–1.12)	n/a
Hypothetical DPC hospital-based analysis (sensitivity analysis)	65/11,278	6.3 (5.0–8.1)	32/6,181	4.9 (3.5–7.0)	0.80 (0.53–1.23)	0.92 (0.60–1.41)	0.81 (0.51–1.30)	0.68 (0.42–1.09)	n/a

CI, confidence interval; PY, person-years; DPC, diagnosis procedure combination; DPP-4, dipeptidyl peptidase-4, SGLT2 = sodium-glucose cotransporter-2.

*Model one was adjusted for age (continuous variable) and sex.

**Model two was adjusted for age (continuous variable), sex, year of drug initiation (continuous variable), medications for hypertension, dyslipidemia, and hyperuricemia.

***Model three was based on an inverse probability weighting of propensity score calculated from age (continuous variable), sex, year of drug initiation (continuous variable), medications for hypertension, dyslipidemia, and hyperuricemia to estimate an average treatment effect.

****Model four was adjusted for age (continuous variable), sex, year of drug initiation (continuous variable), medications for hypertension, dyslipidemia, and hyperuricemia, HbA1c levels, body mass index, and smoking history a complete case analysis. Overall in the JMDC, payer database, 86,681 patients were analyzed and there were 632 outcomes; in a subgroup of new users at clinics, 65,990 patients were analyzed and there were 431 outcomes; in a subgroup of new users at hospitals, 20,691 patients were analyzed and there were 201 outcomes.

Note: the number of outcome events in the hypothetical hospital-based analysis were slightly lower than those in Table 2 because of its different follow-up strategy (for “start or switch to another diabetes drug” and “timing of discontinuation of the initiated drug”) based on only prescriptions in the same hospital initiating the drug.

### 3.5 The hypothetical hospital-based analysis

In the hypothetical hospital-based database study, the incidence rates of the composite outcome (captured in the same hospital where treatment was initiated) was 4.7 (3.8–5.8) and 3.9 (2.8–5.3) among new users of DPP-4 and SGLT2 inhibitors, respectively, suggesting that the incidence rate was underestimated (by nearly half) compared to that estimated in the insurance-based claims data. The crude HR was 0.84 (0.57–1.22) and the aHR in model three was 0.74 (0.49–1.12), suggesting that the point estimates of the HRs were roughly similar (slightly lower), but their confidence intervals were larger than those estimated using the insurance-based claims data. The findings of the sensitivity analysis restricted to DPC hospitals were similar. By each disease outcome, the findings of heart failure outcome were similar to those of the composite outcome, whereas those of stroke and myocardial infarction outcomes showed some fluctuations owing to the smaller number of events (Supplementary Tables S2–S4).

## 4 Discussion

We examined differences in outcome event coverage between insurance-based and hypothetical hospital-based database studies in Japan, and evaluated the impact on pharmacoepidemiologic studies, using diabetes drug use and cardiovascular events as an example. The findings showed that nearly half of cardiovascular events (over half when restricted to DPC hospitals) were captured in the same hospital where the drugs were initiated, leading to underestimation of absolute risks and roughly similar relative risks but wider confidence intervals. At least the point estimates suggested the superior (protective) effect of SGLT2 inhibitors to DPP-4 inhibitors on the risk of cardiovascular events in both insurance-based and hypothetical hospital-based database studies.

Hospital-based databases, consisting of data from individual hospitals with or without standardized formats, are important data sources for pharmacoepidemiology. Compared with insurance-based databases, the strengths of hospital-based databases include availability of electronic health records (including details in patient notes) and examination results in daily clinical practice, such as blood test results and imaging data. However, their major weakness seems to be the lack of traceability of patient visit to other hospitals and clinics, unless linked to other data sources (e.g., insurance-based claims data and follow-up surveys by telephone call). This can cause misclassification of outcome events and (informative) loss-to-follow-up, possibly leading to bias in the study results. Therefore, hospital-based databases may be more suitable for inpatient research (as patients are traceable during hospitalization) than for outpatient research. Nonetheless, hospital-based databases, including those in Japan, are used in outpatient research on common diseases such as diabetes (Kohsaka et al., 2020; Khunti et al., 2021; Kosiborod et al., 2018; Heerspink et al., 2020; Kohsaka et al., 2021; Lam et al., 2021; Goh et al., 2023; Vistisen et al., 2023; Karasik et al., 2023; Sheu et al., 2022; Seino et al., 2021). To our knowledge, the present study is the first to quantify potential biases arising from data fragmentation in hospital-based databases in Japan, using diabetes drugs and cardiovascular events.

As expected, the present study showed that diabetes drugs, as the first choice for type 2 diabetes, were initiated in both clinics and hospitals, reflecting unrestricted access to healthcare in Japan. However, the incidence rates of cardiovascular events were underestimated by nearly half (more than half when restricted to DPC hospitals). When the outcome event coverage is similar between compared groups, relative risks (e.g., HR) remain roughly similar, but confidence intervals become larger owing to the smaller number of outcomes than that in the insurance-based studies.

The differentiation between DPC hospitals and non-DPC hospitals, may affect study results. In Japan, all university hospitals are required to participate in the DPC system, whereas community hospitals participate voluntarily. Although both are acute care hospitals in Japan, DPC hospitals are larger (Yamaguchi et al., 2024), better equipped (Ishimaru et al., 2022), and may be more efficient (Besstremyannaya, 2013) than non-DPC hospitals. These characteristics support our finding that the outcome event coverages in the same hospital were larger when restricted to DPC hospitals, especially for stroke (Supplementary Table S3) and myocardial infarction (Supplementary Table S4). In other words, patients who started drug treatment in non-DPC hospitals were more likely to be transferred to DPC hospitals for stroke and myocardial infarction. This finding may support the use of data from DPC hospitals for better traceability.

The present study has several limitations. First, the generalizability of our results to other diseases is unknown, although cardiovascular events are expected to represent urgent or emergent clinical situations. Second, the JMDC payer database covers individuals aged <75 years, mostly those aged <65 years. It is possible that older people are more or less likely to be transferred to hospitals different from the ones where they initiated drug treatment, compared with younger people. Third, although our primary focus was on the outcome event coverage, instead of a rigorous comparison between DPP-4 and SGLT2 inhibitors for cardiovascular events, the estimated relative risk (aHR) may have been affected by unmeasured and/or residual confounding factors. Previous real-world database studies have concluded that SGLT2 inhibitors are superior to DPP-4 inhibitors in reducing the risk of major adverse cardiac or cerebrovascular events, especially heart failure events (Ng et al., 2025; Kim et al., 2024; D'Andrea et al., 2023; Xie et al., 2023; Rhee et al., 2022; Han et al., 2021; Persson et al., 2018; Filion et al., 2020). The lack of a statistically significant difference in our present study may be due to potential unmeasured and/or residual confounding factors, as well as the small number of outcome events in the relatively younger population in the JMDC payer database. In addition, validity of diagnoses might have affected (diluted) the results, although the small number of outcome events did not allow algorithm creation for outcome definition (e.g., a diagnosis code plus a procedure code or a specific drug treatment), which would have further reduced the number of outcome events. Moreover, we were unable to differentiate admission diagnosis from post-admission diagnosis in the insurance-based claims data; therefore, we assumed that events occurred on the day of hospital admission. Finally, to ensure simplicity and increase comparability in our hypothetical hospital-based analysis, we set the study population and covariate definitions to be the same as those in the insurance-based analysis, whereas the definitions of outcomes and follow-up were based only on the same hospital where drug treatment was initiated. In a genuine hospital-based database study, the study population and covariate definitions may also differ from those in insurance-based database studies, possibly causing additional discrepancies.

In conclusion, through this methodological study of diabetes drugs and cardiovascular events in Japan, we evaluated the difference in outcome event coverage between insurance-based and hypothetical hospital-based database studies. The findings showed that nearly half (more than half when restricted to DPC hospitals) of cardiovascular events were captured in the same hospital where drug treatment was initiated. While outpatient research in Japanese hospital-based databases is possible, researchers and readers should consider the potential limitations arising from limited traceability of patients.

## Data Availability

The datasets presented in this article are not readily available because we obtained data from JMDC Inc. and did not obtain permission to share these data with other parties. Researchers who meet the access criteria can acquire de-identified participant data from JMDC Inc. (https://www.jmdc.co.jp/en/). Requests to access the datasets should be directed to https://www.jmdc.co.jp/en/.
