# Supplementary material for: Difference in outcome event coverage between insurance-based and hospital-based databases: a methodological study of diabetes drug use and cardiovascular events in Japan
Source: Front Pharmacol. 2025 Sep 16;16:1642522. doi: 10.3389/fphar.2025.1642522 (PMC12486410; doi:10.3389/fphar.2025.1642522)
Supplement: Supplementary file 1 [file Table1.docx]

**Supplementary Table S1. Baseline characteristics of participants by type of medical institutions initiating each diabetes drug.**

|  | New users of DPP-4 inhibitors  N = 82,154 | | | | New users of SGLT2 inhibitors  N = 49,562 | | | |
| --- | --- | --- | --- | --- | --- | --- | --- | --- |
|  | Clinics  n = 60,028 | Hospitals  n = 22,126 | P value | SMD | Clinics  n = 35,111 | Hospitals  n = 14,451 | P value | SMD |
| Age, mean (SD) | 54.5 (9.6) | 55.0 (10.2) | <0.001 | 0.050 | 52.1 (9.9) | 53.4 (10.5) | <0.001 | 0.127 |
| Sex, n (%) |  |  | 0.067 |  |  |  | <0.001 |  |
| Men | 43,270 (72.1) | 15,806 (71.4) |  | 0.014 | 25,155 (71.6) | 10,721 (74.2) |  | 0.057 |
| Women | 16,758 (27.9) | 6,320 (28.6) |  | 0.014 | 9,956 (28.4) | 3,730 (25.8) |  | 0.057 |
| Year, n (%) |  |  | <0.001 |  |  |  | <0.001 |  |
| 2009-2013 | 4,194 (7.0) | 1,701 (7.7) |  | 0.027 | 0 (0) | 0 (0) |  | 0 |
| 2014 | 2,294 (3.8) | 962 (4.4) |  | 0.027 | 188 (0.5) | 22 (0.2) |  | 0.065 |
| 2015 | 2,397 (4.0) | 937 (4.2) |  | 0.012 | 331 (0.9) | 91 (0.6) |  | 0.035 |
| 2016 | 3,223 (5.4) | 1,379 (6.2) |  | 0.037 | 762 (2.2) | 238 (1.7) |  | 0.038 |
| 2017 | 4,639 (7.7) | 1,784 (8.1) |  | 0.012 | 1,368 (3.9) | 411 (2.8) |  | 0.058 |
| 2018 | 5,792 (9.7) | 2,127 (9.6) |  | 0.001 | 2,218 (6.3) | 730 (5.1) |  | 0.055 |
| 2019 | 6,419 (10.7) | 2,490 (11.3) |  | 0.018 | 2,975 (8.5) | 1,041 (7.2) |  | 0.047 |
| 2020 | 6,806 (11.3) | 2,469 (11.2) |  | 0.006 | 3,596 (10.2) | 1,285 (8.9) |  | 0.046 |
| 2021 | 7,700 (12.8) | 2,673 (12.1) |  | 0.023 | 5,519 (15.7) | 2,357 (16.3) |  | 0.016 |
| 2022 | 6,717 (11.2) | 2,301 (10.4) |  | 0.025 | 6,292 (17.9) | 3,033 (21.0) |  | 0.078 |
| 2023 | 6,960 (11.6) | 2,343 (10.6) |  | 0.032 | 7,985 (22.7) | 3,634 (25.2) |  | 0.056 |
| 2024 | 2,887 (4.8) | 960 (4.3) |  | 0.023 | 3,877 (11.0) | 1,609 (11.1) |  | 0.003 |
| HbA1c, mean (SD) | 7.5 (1.5) | 7.5 (1.7) | 0.710 | <0.001 | 7.0 (1.4) | 6.7 (1.3) | <0.001 | 0.222 |
| Missing, n (%) | 13,704 (22.8) | 6,230 (28.2) |  | 0.122 | 6,310 (18.0) | 2,970 (20.6) |  | 0.065 |
| BMI, mean (SD) | 26.9 (4.7) | 26.7 (4.8) | <0.001 | 0.042 | 29.3 (5.2) | 28.4 (5.5) | <0.001 | 0.168 |
| Missing, n (%) | 10,575 (17.6) | 4,995 (22.6) |  | 0.124 | 5,056 (14.4) | 2,407 (16.7) |  | 0.062 |
| Smoking history, n (%) |  |  | <0.001 |  |  |  | <0.001 |  |
| Yes | 19,047 (31.7) | 6,748 (30.5) |  | 0.027 | 10,646 (30.3) | 4,194 (29.0) |  | 0.028 |
| No | 30,115 (50.2) | 10,297 (46.5) |  | 0.073 | 19,253 (54.8) | 7,779 (53.8) |  | 0.020 |
| Missing | 10,866 (18.1) | 5,081 (23.0) |  | 0.121 | 5,212 (14.8) | 2,478 (17.2) |  | 0.063 |
| Prescriptions, n (%) |  |  |  |  |  |  |  |  |
| Drugs for hypertension | 30,318 (50.5) | 11,077 (50.1) | 0.260 | 0.009 | 20,216 (57.6) | 10,147 (70.2) | <0.001 | 0.265 |
| Drugs for dyslipidemia | 26,506 (44.2) | 9,459 (42.8) | <0.001 | 0.028 | 17,522 (49.9) | 7,687 (53.2) | <0.001 | 0.066 |
| Drugs for hyperuricemia | 8,763 (14.6) | 3,187 (14.4) | 0.483 | 0.006 | 7,603 (21.7) | 4,099 (28.4) | <0.001 | 0.155 |
| Previous diagnosis history, n (%) |  |  |  |  |  |  |  |  |
| Heart failure | 4,119 (6.9) | 2,749 (12.4) | <0.001 | 0.189 | 4,592 (13.1) | 4,534 (31.4) | <0.001 | 0.451 |
| Stroke | 1,873 (3.1) | 1,223 (5.5) | <0.001 | 0.119 | 995 (2.8) | 700 (4.8) | <0.001 | 0.105 |
| Myocardial infarction | 448 (0.8) | 393 (1.8) | <0.001 | 0.092 | 500 (1.4) | 700 (4.8) | <0.001 | 0.197 |

SD=standard deviation, SMD=standardized mean difference, DPP-4=dipeptidyl peptidase-4, SGLT2=sodium-glucose cotransporter-2.

**Supplementary Table S2. Incidence rates and hazard ratios comparing new users of DPP-4 and SGLT2 inhibitors for heart failure outcome.**

|  | DPP-4 inhibitors | | SGLT2 inhibitors | | Hazard ratio (95% CI) comparing new users of SGLT2 and DPP-4 inhibitors (reference group) | | | | |
| --- | --- | --- | --- | --- | --- | --- | --- | --- | --- |
|  | No. of events  / No. of new users with no history of that outcome | Incidence rate (per 1000 PY) (95% CI) | No. of events  / No. of new users with no history of that outcome | Incidence rate (per 1000 PY) (95% CI) | Unadjusted | Model 1* | Model 2** | Model 3*** | Model 4*** |
| Overall in the JMDC payer database | 358/75,286 | 3.8  (3.4 – 4.2) | 157/40,436 | 3.3  (2.8 – 3.9) | 0.87  (0.72 – 1.05) | 0.99  (0.81 – 1.19) | 0.90  (0.73 – 1.10) | 0.81  (0.66 – 0.99) | 0.95  (0.75 – 1.20) |
| Subgroup analysis of new users at clinics in the JMDC payer database | 220/55,909 | 3.0  (2.7 – 3.5) | 98/30,519 | 2.7  (2.2 – 3.3) | 0.89  (0.70 – 1.13) | 1.02  (0.80 – 1.30) | 0.89  (0.69 – 1.15) | 0.84  (0.65 – 1.08) | 0.91  (0.68 – 1.21) |
| Subgroup analysis of new users at hospitals in the JMDC payer database | 138/19,377 | 6.3  (5.3 – 7.4) | 59/9,917 | 5.2  (4.0 – 6.7) | 0.83  (0.61 – 1.12) | 0.92  (0.68 – 1.26) | 0.88  (0.63 – 1.24) | 0.72  (0.51 – 1.00) | 1.01  (0.68 – 1.50) |
| Hypothetical hospital-based analysis | 65/19,377 | 3.3  (2.6 – 4.2) | 29/9,917 | 2.7  (1.9 – 4.0) | 0.85  (0.55 – 1.32) | 0.92  (0.59 – 1.43) | 0.94  (0.58 – 1.53) | 0.76  (0.47 – 1.22) | n/a |
| Hypothetical DPC hospital-based analysis (sensitivity analysis) | 46/11,991 | 4.1  (3.1 – 5.5) | 25/6,509 | 3.6  (2.5 – 5.4) | 0.92  (0.56 – 1.50) | 1.01  (0.62 – 1.66) | 0.94  (0.54 – 1.62) | 0.75  (0.44 – 1.28) | n/a |

CI=confidence interval, PY=person-years, DPC=Diagnosis Procedure Combination, DPP-4=dipeptidyl peptidase-4, SGLT2=sodium-glucose cotransporter-2.

*Model 1 was adjusted for age (continuous variable) and sex.

**Model 2 was adjusted for age (continuous variable), sex, year of drug initiation (continuous variable), medications for hypertension, dyslipidemia, and hyperuricemia.

***Model 3 was based on an inverse probability weighting of propensity score calculated from age, sex, year of drug initiation, medications for hypertension, dyslipidemia, and hyperuricemia.

***Model 4 was adjusted for age (continuous variable), sex, year of drug initiation (continuous variable), medications for hypertension, dyslipidemia, and hyperuricemia, HbA1c levels, body mass index, and smoking history a complete case analysis. Overall in the JMDC payer database, 89,587 patients were analyzed and there were 391 outcomes; in a subgroup of new users at clinics, 67,850 patients were analyzed and there were 249 outcomes; in a subgroup of new users at hospitals, 21,737 patients were analyzed and there were 142 outcomes.

Note: the numbers of outcome events in the hypothetical hospital-based analysis were slightly lower than those in Table 2 because of its different follow-up strategy (for “start or switch to another diabetes drug” and “timing of discontinuation of the initiated drug”) based on only prescriptions in the same hospital initiating the drug.

**Supplementary Table S3. Incidence rates and hazard ratios comparing new users of DPP-4 and SGLT2 inhibitors for stroke outcome.**

|  | DPP-4 inhibitors | | SGLT2 inhibitors | | Hazard ratio (95% CI) comparing new users of SGLT2 and DPP-4 inhibitors (reference group) | | | | |
| --- | --- | --- | --- | --- | --- | --- | --- | --- | --- |
|  | No. of events  / No. of new users with no history of that outcome | Incidence rate (per 1000 PY) (95% CI) | No. of events  / No. of new users with no history of that outcome | Incidence rate (per 1000 PY) (95% CI) | Unadjusted | Model 1* | Model 2** | Model 3*** | Model 4*** |
| Overall in the JMDC payer database | 244/79,058 | 2.5  (2.2 – 2.8) | 129/47,867 | 2.3  (1.9 – 2.7) | 0.93  (0.75 – 1.15) | 1.08  (0.87 – 1.34) | 1.11  (0.88 – 1.40) | 1.08  (0.84 – 1.40) | 1.18  (0.89 – 1.57) |
| Subgroup analysis of new users at clinics in the JMDC payer database | 166/58,155 | 2.2  (1.9 – 2.6) | 74/34,116 | 1.8  (1.5 – 2.3) | 0.84  (0.64 – 1.11) | 0.99  (0.75 – 1.31) | 1.07  (0.79 – 1.44) | 1.01  (0.74 – 1.38) | 1.14  (0.80 – 1.63) |
| Subgroup analysis of new users at hospitals in the JMDC payer database | 78/20,903 | 3.3  (2.6 – 4.1) | 55/13,751 | 3.5  (2.7 – 4.5) | 1.04  (0.73 – 1.47) | 1.14  (0.81 – 1.62) | 1.06  (0.72 – 1.58) | 1.20  (0.73 – 1.96) | 1.12  (0.69 – 1.83) |
| Hypothetical hospital-based analysis | 31/20,903 | 1.4  (1.0 – 2.0) | 28/13,751 | 1.9  (1.3 – 2.8) | 1.32  (0.79 – 2.20) | 1.47  (0.88 – 2.47) | 1.24  (0.69 – 2.21) | 1.09  (0.62 – 1.91) | n/a |
| Hypothetical DPC hospital-based analysis (sensitivity analysis) | 27/13,196 | 2.2  (1.5 – 3.1) | 24/9,817 | 2.3  (1.6 – 3.5) | 1.08  (0.62 – 1.88) | 1.22  (0.70 – 2.13) | 0.97  (0.52 – 1.84) | 0.89  (0.48 – 1.67) | n/a |

CI=confidence interval, PY=person-years, DPC=Diagnosis Procedure Combination, DPP-4=dipeptidyl peptidase-4, SGLT2=sodium-glucose cotransporter-2.

*Model 1 was adjusted for age (continuous variable) and sex.

**Model 2 was adjusted for age (continuous variable), sex, year of drug initiation (continuous variable), medications for hypertension, dyslipidemia, and hyperuricemia.

***Model 3 was based on an inverse probability weighting of propensity score calculated from age, sex, year of drug initiation, medications for hypertension, dyslipidemia, and hyperuricemia.

***Model 4 was adjusted for age (continuous variable), sex, year of drug initiation (continuous variable), medications for hypertension, dyslipidemia, and hyperuricemia, HbA1c levels, body mass index, and smoking history a complete case analysis. Overall in the JMDC payer database, 98,376 patients were analyzed and there were 256 outcomes; in a subgroup of new users at clinics, 72,535 patients were analyzed and there were 170 outcomes; in a subgroup of new users at hospitals, 25,841 patients were analyzed and there were 86 outcomes.

Note: the numbers of outcome events in the hypothetical hospital-based analysis were slightly lower than those in Table 2 because of its different follow-up strategy (for “start or switch to another diabetes drug” and “timing of discontinuation of the initiated drug”) based on only prescriptions in the same hospital initiating the drug.

**Supplementary Table S4. Incidence rates and hazard ratios comparing new users of DPP-4 and SGLT2 inhibitors for myocardial infarction outcome.**

|  | DPP-4 inhibitors | | SGLT2 inhibitors | | Hazard ratio (95% CI) comparing new users of SGLT2 and DPP-4 inhibitors (reference group) | | | | |
| --- | --- | --- | --- | --- | --- | --- | --- | --- | --- |
|  | No. of events  / No. of new users with no history of that outcome | Incidence rate (per 1000 PY) (95% CI) | No. of events  / No. of new users with no history of that outcome | Incidence rate (per 1000 PY) (95% CI) | Unadjusted | Model 1* | Model 2** | Model 3*** | Model 4*** |
| Overall in the JMDC payer database | 152/81,313 | 1.5  (1.3 – 1.7) | 90/48,362 | 1.6  (1.3 – 2.0) | 1.08  (0.83 – 1.40) | 1.20  (0.92 – 1.57) | 1.20  (0.90 – 1.60) | 1.11  (0.83 – 1.48) | 1.24  (0.90 – 1.72) |
| Subgroup analysis of new users at clinics in the JMDC payer database | 104/59,580 | 1.4  (1.1 – 1.6) | 53/34,611 | 1.3  (1.0 – 1.7) | 0.97  (0.70 – 1.35) | 1.08  (0.77 – 1.51) | 1.04  (0.73 – 1.49) | 1.06  (0.73 – 1.52) | 1.10  (0.74 – 1.63) |
| Subgroup analysis of new users at hospitals in the JMDC payer database | 48/21,733 | 1.9  (1.4 – 2.6) | 37/13,751 | 2.3  (1.7 – 3.2) | 1.25  (0.81 – 1.93) | 1.37  (0.89 – 2.13) | 1.49  (0.91 – 2.44) | 1.14  (0.70 – 1.87) | 1.51  (0.85 – 2.71) |
| Hypothetical hospital-based analysis | 15/21,733 | 0.7  (0.4 – 1.1) | 13/13,751 | 0.9  (0.5 – 1.5) | 1.37  (0.65 – 2.90) | 1.56  (0.74 – 3.32) | 1.70  (0.71 – 4.06) | 1.22  (0.54 – 2.74) | n/a |
| Hypothetical DPC hospital-based analysis (sensitivity analysis) | 13/13,736 | 1.0  (0.6 – 1.7) | 13/9,762 | 1.3  (0.7 – 2.2) | 1.35  (0.62 – 2.92) | 1.50  (0.69 – 3.26) | 1.55  (0.62 – 3.86) | 1.16  (0.50 – 2.73) | n/a |

CI=confidence interval, PY=person-years, DPC=Diagnosis Procedure Combination, DPP-4=dipeptidyl peptidase-4, SGLT2=sodium-glucose cotransporter-2.

*Model 1 was adjusted for age (continuous variable) and sex.

**Model 2 was adjusted for age (continuous variable), sex, year of drug initiation (continuous variable), medications for hypertension, dyslipidemia, and hyperuricemia.

***Model 3 was based on an inverse probability weighting of propensity score calculated from age, sex, year of drug initiation, medications for hypertension, dyslipidemia, and hyperuricemia.

***Model 4 was adjusted for age (continuous variable), sex, year of drug initiation (continuous variable), medications for hypertension, dyslipidemia, and hyperuricemia, HbA1c levels, body mass index, and smoking history a complete case analysis. Overall in the JMDC payer database, 100,242 patients were analyzed and there were 192 outcomes; in a subgroup of new users at clinics, 73,897 patients were analyzed and there were 129 outcomes; in a subgroup of new users at hospitals, 26,345 patients were analyzed and there were 63 outcomes.

Note: the numbers of outcome events in the hypothetical hospital-based analysis were slightly lower than those in Table 2 because of its different follow-up strategy (for “start or switch to another diabetes drug” and “timing of discontinuation of the initiated drug”) based on only prescriptions in the same hospital initiating the drug.
